# Livestock Use on Public Lands in the Western USA Exacerbates Climate Change: Implications for Climate Change Mitigation and Adaptation

**DOI:** 10.1007/s00267-022-01633-8

**Published:** 2022-04-02

**Authors:** J. Boone Kauffman, Robert L. Beschta, Peter M. Lacy, Marc Liverman

**Affiliations:** 1 Department of Fisheries, Wildlife and Conservation Sciences, Oregon State University, Corvallis, OR 97331 USA; 2 Illahee Sciences International, Corvallis, OR 97330 USA; 3 Department of Forest Ecosystems and Society, Oregon State University, Corvallis, OR 97331 USA; 4 Oregon Natural Desert Association, Portland, OR 97211 USA

## Abstract

Public lands of the USA can play an important role in addressing the climate crisis. About 85% of public lands in the western USA are grazed by domestic livestock, and they influence climate change in three profound ways: (1) they are significant sources of greenhouse gases through enteric fermentation and manure deposition; (2) they defoliate native plants, trample vegetation and soils, and accelerate the spread of exotic species resulting in a shift in landscape function from carbon sinks to sources of greenhouse gases; and (3) they exacerbate the effects of climate change on ecosystems by creating warmer and drier conditions. On public lands one cow-calf pair grazing for one month (an “animal unit month” or “AUM”) produces 875 kg CO_2_e through enteric fermentation and manure deposition with a social carbon cost of nearly $36 per AUM. Over 14 million AUMs of cattle graze public lands of the western USA each year resulting in greenhouse gas emissions of 12.4 Tg CO_2_e year^−1^. The social costs of carbon are > $500 million year^−1^ or approximately 26 times greater than annual grazing fees collected by managing federal agencies. These emissions and social costs do not include the likely greater ecosystems costs from grazing impacts and associated livestock management activities that reduce biodiversity, carbon stocks and rates of carbon sequestration. Cessation of grazing would decrease greenhouse gas emissions, improve soil and water resources, and would enhance/sustain native species biodiversity thus representing an important and cost-effective adaptive approach to climate change.

## Introduction

Public lands of the western USA are among the most majestic and biologically diverse landscapes of North America. They are a source of pride and inspiration for the millions of people who visit, recreate, and depend on them, and provide important ecosystem services including clean air and water and vast, unfragmented fish and wildlife habitats and migratory corridors. They also deliver abundant sources of water and other natural resources for agriculture and domestic use. However, the structure and function of these ecosystems are increasingly threatened by the synergistic effects of current land uses and climate change (Remington et al. 2021).

In the coming century, climate change is projected to impact precipitation and temperature regimes worldwide (IPCC 2022), with especially large effects on arid and semiarid landscapes (Palmquist et al. 2016). Predictions for the Intermountain West include increased winter temperatures that will reduce snowpacks and result in earlier spring snowmelt (Barnett et al. 2005; Klos et al. 2014), with important consequences for the amount and timing of soil water recharge (Schlaepfer et al. 2012). In addition, higher temperatures are expected to increase evaporative demand, causing soils to dry out earlier in the year and contributing to longer and drier summer conditions (Palmquist et al. 2016). Shifting patterns of precipitation, increasing temperatures, and rising CO_2_ levels are likely to impact western public lands through alteration of fire regimes and an increased spread of exotic annual grasses (Creutzburg et al. 2015; Mote et al. 2019).

Livestock grazing is the most widespread land use of federally-managed public lands in the western states of the coterminous USA. More than 98 percent of the public lands used for livestock grazing are managed by the Bureau of Land Management (BLM) and the United States Forest Service (USFS) in the western states of the coterminous USA, where a total of 56 million ha and 37 million ha, respectively, are authorized for grazing (GAO 2005; Glaser et al. 2015). This paper focuses on BLM and USFS lands in the western USA where a total of about 93.0 million ha were authorized for grazing (GAO 2005) mostly by beef cattle. However, less than 2.7% of all livestock operators in the USA enjoy the privilege of commercial access to those public lands (Glaser et al. 2015). Rimbey et al. (2015) estimated that only 3.8% of annual livestock forage comes from western US public lands, but this is an overestimate as they only included cows and no other animal type (e.g., bulls, steers). Nor did they account for the increases in beef cattle weights over the past few decades.

**Fig. 1:**
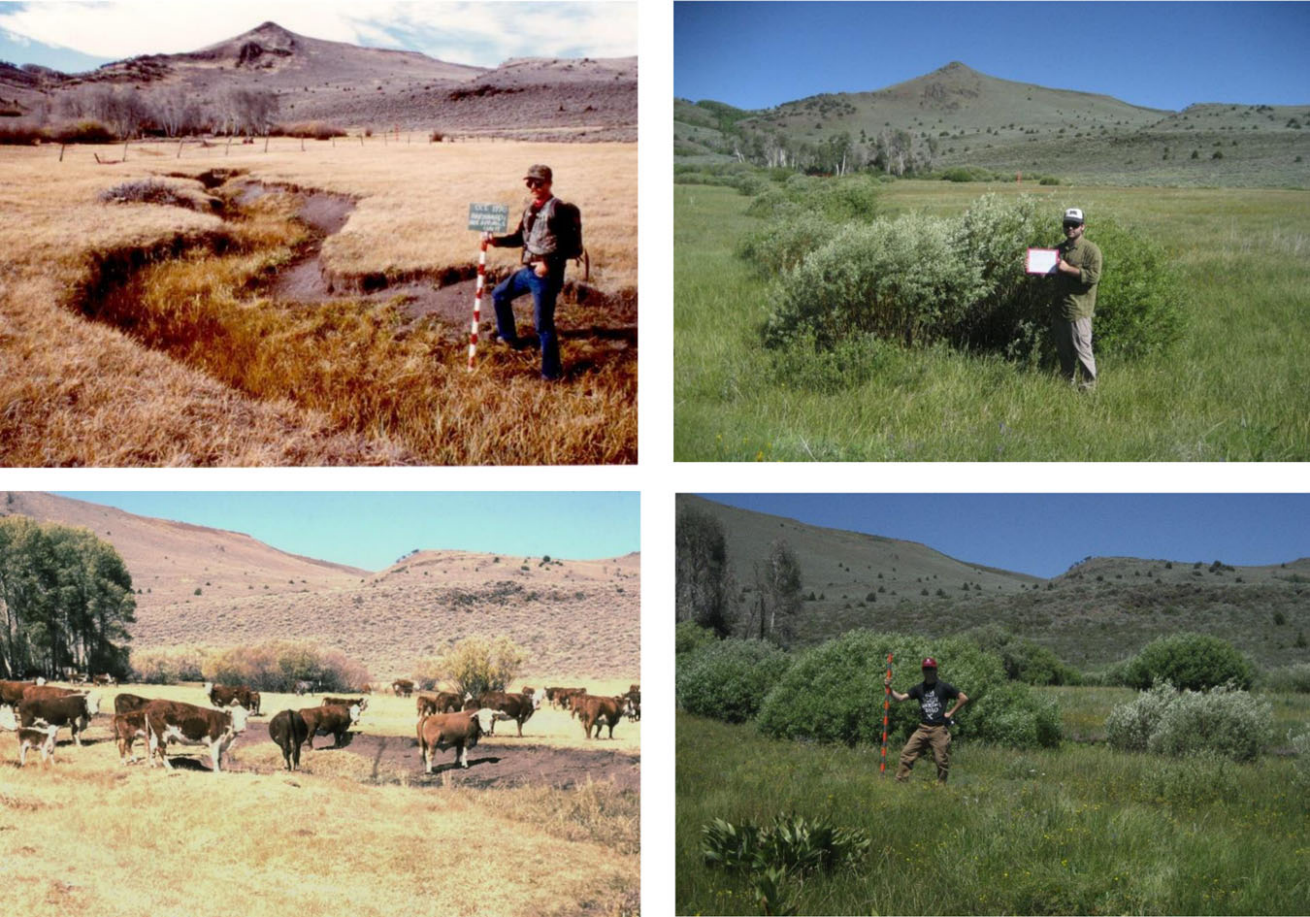
Vegetation change of a riparian ecosystem following cessation of grazing. The left photos are riparian zones on the Hart Mountain National Antelope Refuge, Oregon in 1990 which was the last year of grazing on these public lands. The right photos are the same sites about 24 years after cattle were removed. Wetland vegetation now predominates where there was mostly bare ground and exotic dry grasses. (Photos by W. Pyle and S.Ries)

Animal agriculture is well understood to be a major source of greenhouse gas emissions due to land clearing for pasture, feed production, manure, and the methane emitted by ruminant livestock (Steinfeld et al. 2006). Emissions from livestock production are the largest source of greenhouse gases from the agricultural sector accounting for 72–78% of total agricultural emissions (Gerber et al. 2013; Springman et al. 2018), and cattle are the dominant ruminant grazing animal producing emissions in the USA and globally (UNEP 2021). Livestock generate more greenhouse gases than the entire transportation sector (Steinfeld et al. 2006). Livestock grazing has also resulted in widespread vegetation and soil degradation including reductions in biological diversity, carbon stocks, net primary productivity, and soil nutrient contents (Kauffman and Pyke 2001; Kauffman et al. 2009; Kauffman et al. 2016). The effects of climate change will likely be exacerbated by livestock (Fig. 1; Beschta et al. 2012).

Because the largest proportion of greenhouse gases produced by the agricultural sector comes from the methane emissions and land use related to livestock production (Lazarus et al. 2021), natural resource agencies and the agricultural sector should address these sources of emissions. Given the innumerable resource and social values associated with public lands, coupled with their relatively low production value for livestock, these areas represent a logical focal point for reducing greenhouse gas emissions in a socially and economically effective manner.

Federal public lands in the Western USA span diverse expanses of forests, shrublands, and grasslands, nearly all of which are grazed by domestic livestock annually. We focus on the interactions of grazing in the sagebrush biome which contains landscapes dominated by diverse assemblages of shrublands, woodlands, grasslands and riparian wetlands. Sagebrush-dominated ecosystems are the most extensive semiarid vegetation type in the western USA, comparable in size to the Great Plains or the eastern deciduous forests (Neilson et al. 2005; Austin et al. 2019). Sagebrush now occupies an estimated 651,316 km^2^ over portions of 14 western States (Remington et al. 2021). The sagebrush ecosystem is also among the most vulnerable to loss or degradation in North America (Miller et al. 2011; Chambers and Wisdom 2009). The most widespread dominant species in this varied biome is big sagebrush (*Artemisia tridentata*). Of the big sagebrush-dominated ecosystems, the Wyoming big sagebrush (*A t. wyomingensis*) is the most xeric and widespread of the subspecies. Other abundant big sagebrush subspecies dominated ecosystems include Basin big sagebrush (*A t. tridentata*) and Mountain big sagebrush (*A.t. vaseyana*).

The first objective of this paper was to review the role that public lands of the sagebrush biome in the western USA—by far the largest biome in the West—could serve in addressing the climate and extinction crisis. We did this by examining (1) the degree to which cattle and associated management exacerbate the effects of a warming and drying climate in this vast biome and (2) the degree to which cattle cause these sagebrush landscapes to shift from significant carbon sinks to significant sources of greenhouse gases. Then, moving beyond the sagebrush biome, our second objective was to undertake a meta-analysis using animal use and enteric and manure emissions data from US and international agencies to determine the importance of cattle grazing on public lands of the western USA as sources of greenhouse gases, and the social costs associated with these emissions.

To examine carbon stock losses associated with conversion of native ecosystems to exotic-dominated grasslands [e.g., annual dominance of cheatgrass (*Bromus tectorum*) or perennial dominance by crested wheatgrass (*Agropyron cristatum*)] we calculated mean aboveground carbon stocks of sagebrush, woodlands, and grasslands from literature values (Supplementary Information Table S1). In order to determine potential greenhouse gas emissions from livestock use on public lands, we conducted a meta-analysis combining datasets of quantities of animal use, emissions from individual animals and the social costs of greenhouse gases coming from cattle.

## Cattle Grazing Exacerbates the Effects of Climate Change

**Fig. 2:**
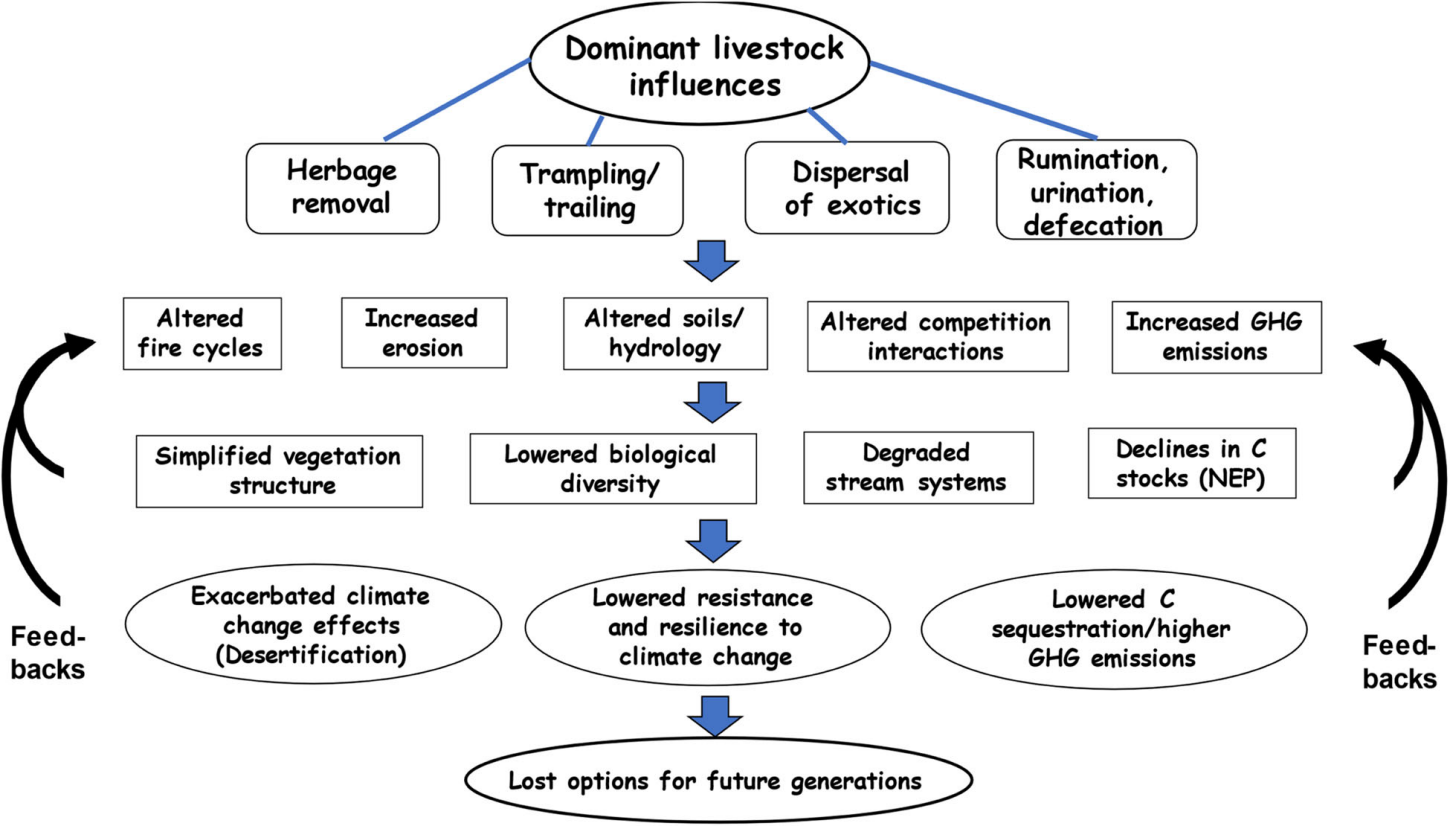
The interacting effects of livestock grazing and climate change on western rangelands. There are four primary immediate effects of livestock: herbage removal, trailing trampling effects, dispersal of exotics, and creation of metabolic and nonmetabolic waste products. Through time, these effects on native rangelands affect fire regimes, increase erosion, compact soils affecting ecosystem hydrology, and alter competitive relationships between plant species. These actions decrease the net ecosystem productivity (NEP) such that the rangelands shift from carbon sinks to net sources of greenhouse gases. Products of animal metabolism are significant additional sources of greenhouse gases, especially CH_4_ and N_2_O. Ultimately the results of grazing have led to a simplification of vegetation structure typified by increases in exotic, ruderal, and less palatable species, that are more adapted to the drier conditions created by lower water holding capacities of compacted soils. The shifts in species composition further decrease the capacity of rangeland ecosystems to function as carbon sinks. Other impacts of grazing include a decline in riparian vegetation structure, shifts to drier species dominance, and degraded stream channels which increase stream temperatures, ground surface temperatures and alter stream flows. The consequent shifts in the net ecosystem productivity of the landscape, coupled with GHG additions from livestock, results in additional contributions to the greenhouse gases causing climate change. The effects of livestock accentuate the effects of climate change such as increased stream and air temperatures, loss in biological diversity, and an overall decline in the productivity of rangelands (desertification). There are also strong feedbacks associated with climate change. The warmer and drier temperatures, and reduced snow pack associated with climate change interacts with livestock grazing to negatively affect stream flows, water quality and biological diversity. These factors result in further degradation and a lower capacity for carbon storage, hence higher greenhouse gas emissions

Regardless of season of use or grazing intensity, domestic livestock generally influence ecosystems in four direct ways: (1) by removing vegetation through grazing; (2) by trampling soils, biotic soil crusts, streambanks and vegetation; (3) by redistributing nutrients via defecation and urination; and (4) by dispersing or creating favorable conditions for the establishment and dominance of exotic organisms, including noxious plant species and pathogens (Fig. 2; Fleischner 1994; Belsky et al. 1999; Dwire et al. 1999). Grazing by livestock will directly reduce the quantity and quality of available forage for wild grazers while modifying habitat quality for numerous wildlife species. Livestock herbivory also decreases the protective litter layer and the quantity of organic matter (and carbon) that can be incorporated into soils. Physical damage through trampling occurs from soil compaction and physical damage to biotic soil crusts and vegetation. Defecation and urination, especially in riparian zones and near stream channels, can have serious consequences for water quality and aquatic organisms. Feces and rumination also result in production of methane and nitrous oxide. Finally, livestock are vectors for the spread of exotic species and create conditions for their establishment. Grazing spreads invasive annual grasses by removing native perennial grasses (Reisner et al. 2013; Rosentreter 1994; Chambers et al. 2007; Belsky and Blumenthal 1997), by disturbing soils (Olff and Ritchie 1998), and by damaging biological soil crusts (Belnap 2006; Chambers et al. 2014; Reisner et al. 2013; Ponzetti et al. 2007; Warren and Eldridge 2001; Belnap 1995). Livestock also distribute annual grass seeds across the landscape through their hooves, fur, and digestive tracts (Schiffman 1997; Olff and Ritchie 1998; Chambers et al. 2014; Mack 1981; Knapp 1996). Unlike the bunchgrasses native to the Intermountain West and Pacific Northwest of the USA, many exotic plant species that have appeared or proliferated since the introduction of livestock in the mid-nineteenth century evolved under continuous grazing pressure and are well adapted to the disturbed conditions caused by livestock grazing (Mack and Thompson 1982).

These four primary livestock influences interact to result in significant physical and biotic alterations of ecosystem structure and function. Among other shifts in ecosystem structure and function, alterations include modified fire cycles, increased soil erosion, lowered water holding capacities, and decreased infiltration rates in soils (Dwire et al. 1999; Kauffman and Pyke 2001).

The cumulative effect of long-term domestic livestock use of public lands typically results in simplified vegetation and soil structure, dominance of exotic annual plant species, degraded riparian zones and aquatic ecosystems, and lowered carbon stocks (Fig. 2). These effects contribute to desertification, a lowered resistance to the stresses associated with a changing climate, a shift from net carbon sinks to sources of greenhouse gases, biotic impoverishment, and the loss of ecosystem services provided by native plant communities. Further, there are strong reinforcing feedback loops between livestock grazing and climate change. For example, decreased vegetation structure, root mass, and soil organic matter can result in less sequestration of methane (Tang et al. 2013), lowered carbon stocks (Meyer 2011), and less water stored due to declines in water holding capacity (Kauffman et al. 2004). In addition, the loss of deep-rooted sagebrush and other shrub species by fire, overgrazing, or purposeful conversion to exotic grasslands would reduce biotic access to deep soil water which exacerbates climate change effects (Franklin and Dyrness 1973; Rau et al. 2011).

The loss of vegetation structure associated with declines in deciduous woody species in riparian zones, such as palatable quaking aspen (*Populus tremuloides)* and willows (*Salix* spp.), due to herbivory and trampling by livestock, results in warmer microclimates and lower soil water holding capacities, thus exacerbating the warming and drying effects of climate change (Beschta et al. 2012; Kauffman et al. 2022). Furthermore, increased levels of carbon dioxide in arid shrubland ecosystems favor exotic annual grasses at the expense of native vegetation (Mooney and Hobbs 2000).

The cumulative effects of livestock grazing coupled with climate change in semiarid landscapes of the Intermountain and Pacific Northwest of the USA represent lost options for future generations, including losses in biodiversity and clean water, as well as the spiritual, social, recreational, and sustainable economic opportunities these public lands can provide (Fig. 2).

### Livestock Grazing Degrades Riparian Zones and Wetlands

Although riparian areas and wetlands cover less than 1–2 % of the western USA landscape, their ecological significance far exceeds their limited physical area (Elmore and Beschta 1987; Kauffman and Krueger 1984). They are highly productive and ecologically valuable due to the vital habitats they provide and their importance to aquatic ecosystems (Kauffman et al. 2001; Fleischner 1994). They are also significant carbon sinks. Wetlands, including riparian zones, are among the largest carbon stocks of any plant community in North America, especially in semi-arid zones. Nahlik and Fennessy (2016) reported that soils of palustrine/riverine wetlands of western USA wetlands stored 236 Mg C ha^−1^. These stocks are about 3 to 6 times that of upland forests of eastern Oregon (≈61 Mg C ha^−1^; Law et al. 2018).

Livestock grazing has been found to exacerbate the effects of climate change in riparian ecosystems, leading to warmer and drier conditions in these vital habitats. In a broad study of riparian composition in eastern Oregon, Kauffman et al. (2022) found the abundance of wetland-obligate native sedges (*Carex* spp.) and broad-leaved forbs were significantly greater in ungrazed areas. In contrast, exotic species adapted to grazing, such as Kentucky bluegrass (*Poa pratensis*) and white clover (*Trifolium repens*), were more abundant in grazed stream reaches. However, following cessation of livestock grazing, facultative- and obligate-wetland species replaced ones adapted to drier environments (Kauffman et al. 2022).

Livestock removal has been found to result in significant recovery of soil, hydrological, and vegetation properties of riparian ecosystems that, at watershed scales, can mediate climate change stresses on stream channel morphology, water quality, and the aquatic biota. For example, Kauffman et al. (2004) estimated that under saturated conditions, the pore space measured in wet-meadow communities excluded from livestock grazing would contain 121,000 l ha^−1^ (121 Mg ha^−1^) more water in only the surface 10 cm of soil than those in grazed wet-meadow communities.

### Livestock Grazing Decreases the Sequestration and Storage of Carbon

The total aboveground carbon stocks in sagebrush-dominated communities range from about 2.7 Mg C ha^−1^ for Wyoming big sagebrush to 7.8 Mg C ha^−1^ for Basin big Sagebrush. The aboveground carbon stocks of western juniper (*Juniperus occidentalis*) dominated woodlands are ≈18.3 Mg C ha^−1^, increasing to about 97 Mg C ha^−1^ for interior coniferous forests (Supplementary Information, Table S2; Law et al. 2018). Degradation of native plant communities to exotic annuals or purposeful type conversion by the seeding of exotic perennial grasses, results in carbon losses (Bradley et al. 2006; Rau et al. 2011; Nagy et al. 2020). The mean aboveground carbon stocks for converted stands were 0.5 Mg C ha^−1^ for crested wheatgrass seedings and 0.23 Mg C ha^−1^ for cheatgrass-dominated stands. Comparing these losses to the most abundant and most xeric of big sagebrush communities (Wyoming big sagebrush) suggests at least an 88% decline in aboveground biomass when they are converted to a cheatgrass-dominated sites and an 84% decline when converted to crested wheatgrass. These losses do not reflect the additional losses coming from declines in soil carbon stocks that would occur with the extirpation of deep-rooted shrubs and grasses (Meyer 2011; Rau et al. 2011).

**Fig. 3:**
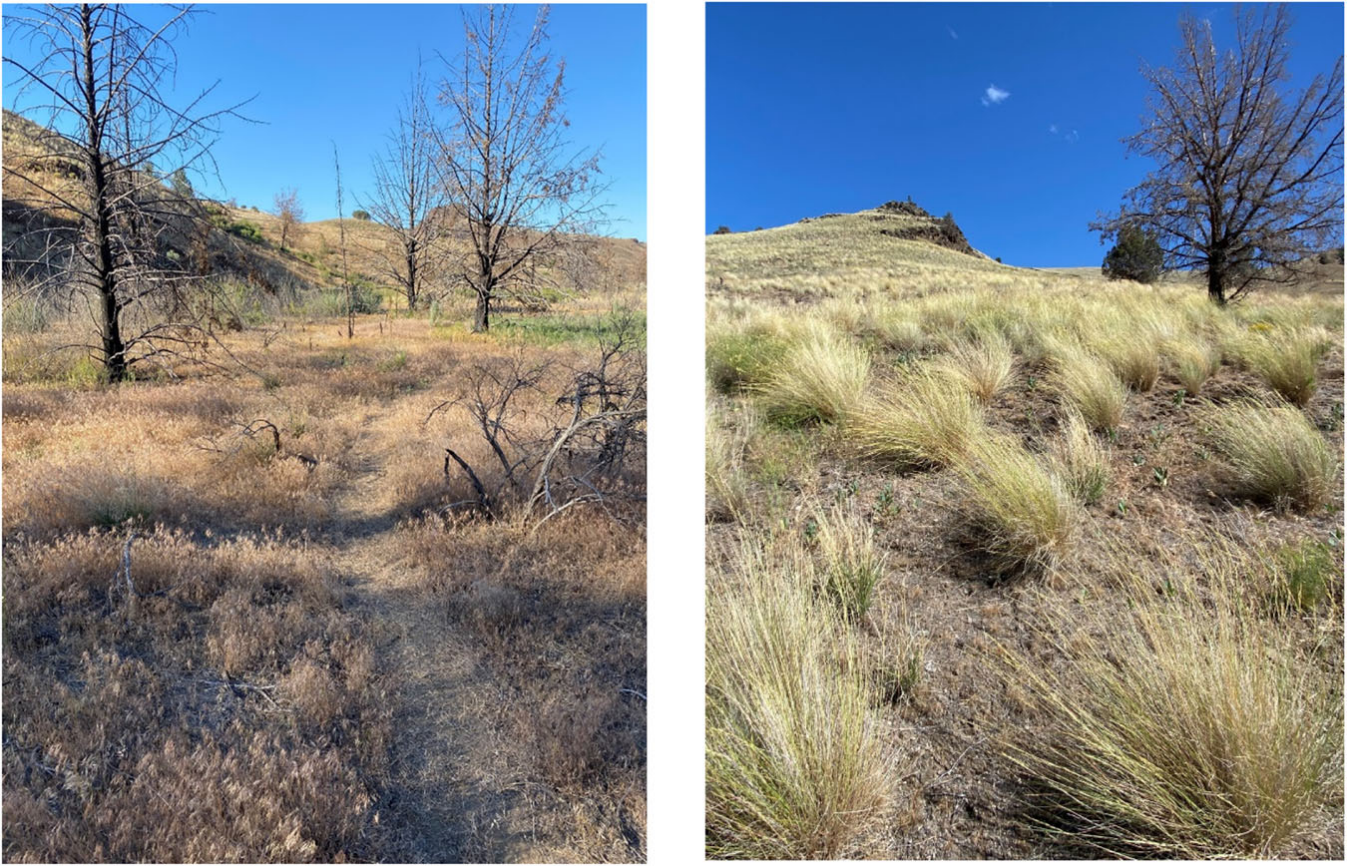
Left photo: A long-term grazed site dominated by the annual exotic Cheatgrass (*Bromus tectorum*), Prineville District, BLM, Oregon. In addition to a dominance by exotic species, there is an absence of biotic soil crusts. The site had been burned about three years prior to the time this photo was taken. Right photo: An ungrazed site dominated by native species, Prineville District, BLM, Oregon. The dominant grasses are Bluebunch Wheatgrass (*Pseudoregnaria spicata*). The interspaces are dominated by native forbs, Sandberg’s Blue grass (*Poa sandbergii*) and biological soil crusts. Exotic annuals are <1% cover that this site. This site had also burned ≈3 years prior to the taking of this photo (Photos by J.B. Kauffman)

Cheatgrass exhibits various attributes that makes it extremely tolerant of even highly intensive grazing (Reisner et al. 2013). The expansion of cheatgrass across much of the western USA associated with livestock grazing has long been known (Franklin and Dyrness 1973; Mack and Thompson 1982), but its implications on carbon cycling have been overlooked (Bradley et al. 2006; Meyer 2011). Livestock grazing exacerbates cheatgrass dominance in sagebrush-dominated ecosystems by adversely impacting key mechanisms mediating resistance to invasion (Reisner et al. 2013). This includes losses of biotic soil crusts due to trampling as well as excessive herbivory of grazing-sensitive native bunchgrasses, decreasing their capacity to compete with the exotic annuals. The loss of biotic soil crusts and other aggregated soil surface conditions have several important ecological ramifications because they: (1) inhibit erosion (Belnap 2006); (2) are an important source of nitrogen fixation in sagebrush steppe ecosystems; (3) serve as natural fire breaks, especially in low elevation sagebrush habitats where they can cover over 40% of the soil surface (Rosentreter 1986); and (4) inhibit cheatgrass germination (Reisner et al. 2013; Fig. 3).

Williamson et al. (2020) reported that increased cheatgrass occurrence and prevalence corresponded with livestock grazing regardless of variation in climate, topography, or community composition, and their results provide no support for a hypothesis that contemporary grazing regimes, or grazing in conjunction with fire, can suppress cheatgrass. Meyer (2011) concluded the elimination of perennial understory vegetation and biotic soil crusts were a nearly inevitable consequence of livestock grazing western shrublands, thus opening these systems to annual grass invasion, altered fire regimes, and loss of a major carbon sink. After examining the causes of cheatgrass invasion, Reisner et al. (2013) concluded that if the goal is to conserve and restore resistance of these sagebrush ecosystems, managers should consider maintaining or restoring: (1) high bunchgrass cover and structure characterized by spatially dispersed bunchgrasses and small gaps between them; (2) a diverse assemblage of bunchgrass species to maximize competitive interactions with cheatgrass in time and space; and (3) biological soil crusts to limit cheatgrass establishment. Cessation of livestock grazing is a passive restoration approach that eliminates cumulative effects of cattle use and may well be the most effective means of reducing the degradation of biological diversity of public rangelands where cheatgrass and other exotics are currently prevalent.

**Fig. 4:**
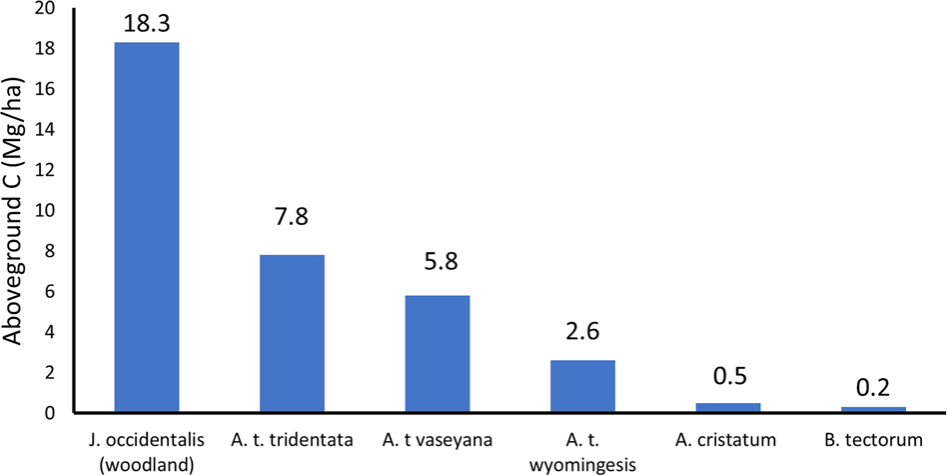
Total aboveground carbon stocks for non-forested ecosystems occupying public lands of the intermountain West. Total aboveground carbon stocks range from 2.69 (Mg C ha^−1^) for Wyoming Big sagebrush (*A.t wyomingensis*) communities to 7.8 Mg C ha^−1^ for Basin big sagebrush (*A. t. tridentata*) stands. The aboveground carbon stocks of intermountain woodlands are 18.3 Mg C ha^−1^ and for coniferous forests is about 97 Mg C ha^−1^ (Law et al. 2018). In contrast, mean aboveground carbon stocks for converted stands were 0.5 Mg C ha^−1^ for crested wheatgrass (*Agropyron cristatum*) and 0.2 Mg C ha^−1^ for cheatgrass (*Bromus tectorum*) stands. There is an 84% decline in aboveground biomass when Wyoming Big sagebrush stands are converted to crested wheatgrass and an 88% decline when they are converted to cheatgrass

There were at least 12.7 million ha of land dominated by cheatgrass in 2000 (Zouhar 2003). Conservatively using mean aboveground carbon stock estimates for Wyoming big sagebrush (2.6 Mg C ha^−1^ and for cheatgrass (0.2 Mg C ha^−1^; Fig. 4) suggests that by 2000 there was a carbon loss equivalent to at least 111.8 Tg CO_2_e due to conversion of native rangelands to cheatgrass in this biome alone.

In addition to livestock grazing, many other proposed vegetation management activities associated with livestock management will also likely shift rangeland ecosystems from net sinks of atmospheric carbon to net sources of greenhouse gases. These include type conversion through seeding exotic grasses, removing native juniper trees, and constructing large-scale networks of fuel breaks (Jones 2019).

Crested wheatgrass is a nonnative perennial grass species that public land managers continue to seed in an attempt to stabilize landscapes following fire and to facilitate livestock grazing. In the USA, it was first planted in 1898 and gained wide acceptance in the 1930s (Zlatnik 1999). However, there is a growing body of research that suggests crested wheatgrass alters rangeland sites in ways that exacerbate climate change. Seeding a disturbed site with crested wheatgrass may prohibit the establishment of native species and the return to pre-disturbance plant structure and diversity (Zouhar 2003; Zlatnik 1999). Soils in crested wheatgrass stands often have higher bulk density, fewer water stable aggregates, and lower levels of organic matter and nitrogen compared to soils native grass-dominated stands. Dormaar et al. 1995 found that crested wheatgrass seedings could neither return nor maintain the chemical quality of the soils in relation to that of the native rangeland. Crested wheatgrass seedings result in lower water holding capacity and lower nutrient and carbon storage than the native communities they replaced. The continued conversion of native ecosystems and planting of crested wheatgrass or other exotic species is ill advised (Lesicu and DeLuca 1996).

Conversion of native sagebrush grasslands to crested wheatgrass seedings contributes to climate change through a substantial decrease in carbon stocks. The mean carbon stock of Wyoming big sagebrush stands is 2.6 Mg C ha, and for converted stands dominated by the introduced crested wheatgrass, it is 0.5 Mg C ha (Fig. 4; Supplementary Information, Table S1). Crested wheatgrass seedings have been established on 3.2 to as much as 10.4 million ha in North America (Zouhar 2003). Conservatively using the mean aboveground carbon stock of Wyoming big sagebrush as the pre-seeding mass, the carbon losses are estimated to total 24.7 to 80.2 Tg CO_2_e through this conversion.

## Cessation of Livestock Grazing Increases Carbon Storage

Cessation of grazing is an effective means of increasing carbon storage in both riparian zones and uplands (Fig. 1) as both aboveground and belowground carbon stocks increase with ecosystem recovery. In the western USA, riparian areas and wetlands are focal points for carbon sequestration. Although they cover only 1–2% of the landscape, stream and riparian areas exert an outsized influence on ecosystem function and biodiversity. For example, over a 10-year period of livestock exclusion, surface soils (0–10 cm depth) in ungrazed riparian zones of eastern Oregon sequestered an additional 12.5 Mg C ha^−1^ in dry meadows and 28.5 Mg C ha^−1^ in wet meadows compared to paired grazed sites (Kauffman et al. 2004)

There is also a significant accumulation in root mass following the cessation of livestock grazing, which is a critical influence on stream channel structure as well as carbon sequestration. Kauffman et al. (2004) reported that 10 years of rest from livestock grazing resulted in an increased root mass of 2.1 Mg C ha^−1^ in dry meadows and 4.3 Mg C ha^−1^ in wet meadows (assuming a root carbon concentration of 39%; Kauffman and Donato 2012). Combining differences in root mass and soil organic matter suggests that ungrazed sites have increased carbon sequestration rates of 1.5 Mg C ha^−1^ year^−1^ in dry meadows and 3.3 Mg C ha^−1^ year^−1^ in wet meadows (5.4 and 12.0 Mg CO_2_e ha^−1^ year^−1^, respectively) over that of grazed riparian zones.

The quantity of carbon that would be sequestered in the absence of livestock is a sacrificed benefit in favor of livestock grazing. Using the mid-point values of the additional soil and root carbon sequestration from wet and dry riparian meadows through rest from livestock grazing (2.4 Mg C ha^−1^ year^−1^; Kauffman et al. 2004), and conservatively assuming only 1% of the grazed BLM and USFS public lands in the 11 western states are occupied by riparian zones and other wetlands (about 930,000 ha), an additional 2.2 Tg C year^−1^ (8.1 Tg CO_2_e year^−1^) of carbon could be sequestered through cessation of livestock grazing in riparian areas alone. Furthermore, cessation of grazing would improve riparian plant functions such as streambank stabilization and stream cover, and hence cooler water temperatures vital to fish and other aquatic species.

Net ecosystem carbon balance (NECB) is the net rate of C accumulation or loss in ecosystems and is important in ascertaining their role as functional carbon sinks or sources of greenhouse gases (Chapin et al. 2006). Although few studies have reported NECB in sagebrush ecosystems, Gilmanov et al. (2006) reported net ecosystem carbon gains of 0.2 Mg C year^−1^ for Wyoming big sagebrush (Oregon) and 0.7 Mg C year^−1^ for three-tip sagebrush (*Artemisia tripartita*) (Idaho). Comparing the riparian zones to uplands suggest that while riparian zone only cover about 1–2% of the landscape they may potentially account for 3–18% of the carbon gain in sagebrush landscapes. The 18% estimate assumes riparian zones occupy 2% of the landscape and the NECB of uplands carbon stocks are those of Wyoming big sagebrush.

### Livestock Grazing Will Exacerbate the Effects of Fire in a Changing Climate

Fire seasons in the western USA now average 78 days longer than in 1970, and future climate change could lengthen the period of annual extreme fire-weather conditions (Abatzoglou and Williams 2016). An elevated wildfire occurrence in concert with the current levels of livestock use will likely facilitate an increase in the degradation of sagebrush and other native shrub-perennial grass communities and their conversion to plant communities dominated by exotic grasses (D’Antonio and Vitousek 1992). These will have positive feedbacks accelerating climate change (Fig. 2) through increasing greenhouse gas emissions while diminishing the size of ecosystem carbon sinks.

There is a strong synergism between cheatgrass, fire, and livestock grazing. Cheatgrass is well known to increase following fire in grazed rangelands (Fig. 3A, Zouhar 2003). However, in ungrazed ecosystems native vegetation typically dominates following fire and cheatgrass invasion has been low to non-existent (Fig. 3B). This pattern of native species resilience following fire in ungrazed landscapes has been reported in bunchgrass prairies (Montana; Antos et al. 1983), Wyoming big sagebrush (Oregon and Washington; Ellsworth et al. 2016; Reis et al. 2019; Ponzetti et al. 2007), Mountain big sagebrush (California and Oregon, Ellsworth and Kauffman 2010; Ellsworth and Kauffman 2017), and Basin big sagebrush ecosystems (Oregon; Ellsworth et al. 2020). Furthermore, many native grasses and forbs that are key species in springtime diets of greater sage-grouse (*Centrocercus urophasianus*) exhibit high rates of reproduction following fires (i.e., fire-enhanced flowering) and in the absence of livestock grazing and trampling (Wroblesky and Kauffman 2003).

### Home on the Range Where the Deer and Antelope Get 8%

**Fig. 5:**
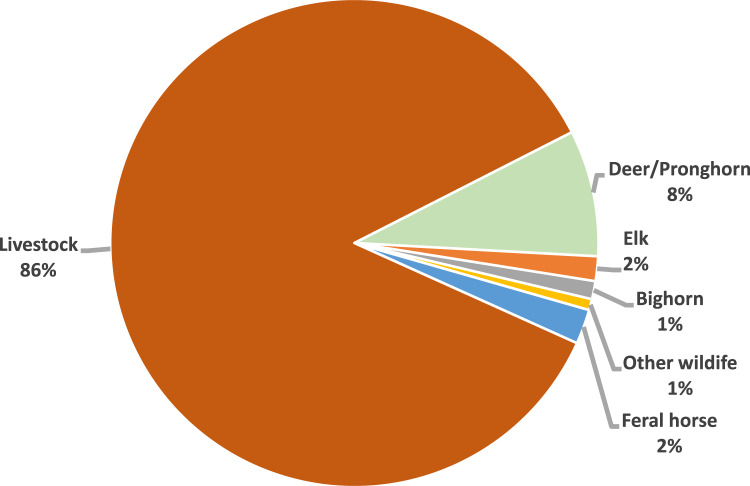
Forage allocation for domestic livestock, feral horses, and wildlife on the Bureau of Land Management (BLM) Lakeview District, Oregon (USDI BLM 2003a)

Examination of forage allocation on public lands suggests that management is strongly skewed towards livestock production at the expense of other uses especially wildlife and the sustainability of the inherent biological diversity of the land. For example, in the Lakeview, Resource Management Plan (USDI BLM 2003a), which guides land and resource management on about 1.3 million ha of BLM-managed public land in Lake and Harney counties in southeastern Oregon, cattle were allocated 81% of the forage (Fig. 5). Deer and antelope were allocated 8% of the forage. Further, there are about 363 species of wildlife that utilize public lands in Southeast Oregon (Thomas et al. 1979; Kauffman et al. 2001; Kauffman and Krueger 1984; USDI BLM 2003b) and they were allocated only 1% (Fig. 5). These wildlife species provide a number of ecosystem services to people and society including commodity/utilitarian values, ecological process values, recreational values, esthetic values, cultural values and educational values.

### While Animal Science Has Advanced, Range Management Has Not

Livestock use on public lands is measured in animal unit months (AUMs); a term developed more than a century ago (Smith 2017). An AUM is defined as the amount of forage required to feed one 1000 lb. (454 kg) cow and calf for one month (Heady 1975; Smith 2017). But the average cattle weight today is significantly greater than 454 kg. The US Environmental Protection Agency (USEPA 2018) reported the mean weight of a cow was 554 kg (1221 lbs) in 1990 and 611 kg (1348 lbs) in 2015. Thus, the same number of domestic animals (cows) on public lands over time represents a *de facto* increase in overall forage use and physical influences (Heady 1975; Smith 2017). Based upon the metabolic weight of modern cattle, a single cow and calf in 2021 would account for ≈1.25 AUMs. Yet, this increase in cattle weight and associated influences (greater feed intake, greater physical damage) are not currently considered in forage allocations, carrying capacities, or stocking rates. If the increase in the average size of cattle were included, the AUMs counted on public lands may have actually increased by 25% over the past two decades.

In 2015, there were about 29 million head of beef cattle in the US (US Department of Agriculture National Agricultural Statistics Service 2021; USEPA 2018) and the mean weight of a cow was 611 kg for that year. Thus, there were 441 million AUMs of forage required for USA beef cows alone. The 14.1 million AUMs arising from western public lands provide about 3.2% of the forage used by all cows in the USA, which is similar to the estimate of 3.8% reported by Rimbey et al. (2015) (Supplementary Table S5). However, this estimate does not account for other types of beef cattle such as bulls, steers, and replacement heifers. Including all beef cattle (except calves) suggests that the total AUMs of forage used by the USA cattle population was ≈860 million AUMs. Therefore, public lands actually provide <1.6% of all forage consumed by beef cattle in the USA.

The grazing practices employed on public lands have changed little over the last century. Common grazing practices such as deferred rotational grazing were first recommended by Arthur Sampson in 1913-14 (Heady 1975), and rest-rotation grazing was developed in the late 1950s (Stoddart et al. 1975). Given the climate changes occurring in the western USA, the grazing systems currently being utilized may no longer have the desired effects they were intended to achieve. For example, the theory behind grazing early in the growing season is that it would allow vegetation to recover through replenishment of stored carbohydrates via regrowth. By removing livestock before most spring and summer precipitation occurs, it was assumed plants would be able to store carbohydrates, set seed, and maintain their vigor (USDI BLM 2003b). But climate change is projected to result in drier summer conditions (Palmquist et al. 2016) where soil moisture will not be available for regrowth. This will affect native plants to a much greater extent than exotic annuals. Thus, spring grazing under conditions of limited soil moisture would exacerbate the effects of climate change on the native flora.

Climate change may also result in lowered suitability of public lands as grazing resources during dormant seasons. In the future, forage quality during summer through the winter months will be lower because of warmer and drier conditions, as well as expected increases in the abundance of exotic annuals. A decrease in forage quality (higher in fiber and lower in digestible energy) will result in a higher emissions intensity (kg of enteric methane emitted per kg of animal gain) from cattle as they increasingly consume poorer quality forage. In addition, with warmer winter conditions and less snow cover it can be assumed that soils will not be frozen and thus will be prone to increased compaction via livestock trampling. This trampling damage would exacerbate the effects of climate change through decreased water holding capacity (Kauffman et al. 2004).

## Public Lands Are Sources of Greenhouse Gas Emissions Arising from Livestock Grazing and the Social Cost Is Significant

In this section, we determined greenhouse gas emissions attributed to enteric fermentation and manure deposition originating from cattle grazing the public lands in the western USA. We assumed that AUMs represented cow-calf pairs, although yearling steers grazed at the same stocking level would likely produce similar results.

The relative capacity of a greenhouse gas to trap heat in the global climate system over a given time frame, compared to that of carbon dioxide, is expressed as its global warming potential (GWP). The GWP of methane (with climate-carbon feedbacks) is 86 over a 20-year interval (GWP-20) and 34 for a 100-year interval (GWP-100; IPCC 2013). Nitrous oxide, arising from manure deposition has a GWP of 268 and 298 at 20- and 100-year intervals, respectively (IPCC 2013). Because methane has a comparatively short lifetime in the atmosphere, strategies to reduce methane emissions from livestock provide an opportunity to arrest the rate of anthropogenic global warming more rapidly than strategies focused on reduction of carbon dioxide emissions. Based on the urgent need to reduce methane emissions to avoid catastrophic tipping points in the climate system during the next 15–35 years, Howarth (2014) suggested the 20-year GWP was more relevant than the 100-year GWP. In this section we report both the 20- and 100-year GWPs for identifying the potential greenhouse gas emissions associated with public lands livestock grazing.

GHG emissions were determined using three different approaches. For the first two approaches (20-year and 100-year GWP), the USEPA (2018) national default values for beef cattle were used to calculate the emissions from public lands grazing. This is 95 kg methane year^−1^ for cows and 11 kg methane year^−1^ for calves. Therefore, one cow-calf pair would emit 106 kg methane year^−1^ from enteric fermentation (Supplementary Information, Table S2). To determine methane and nitrous oxide emissions from manure deposition, default values from the IPCC (2006) were used.

The third approach (IPCC default) used global default values of methane emissions from enteric fermentation for beef cattle (IPCC 2006). Methane emissions from enteric fermentation are 53 kg animal^−1^ year^−1^ (Supplementary Information, Table  S2). Unlike the USEPA (2018) estimates, these emission values do not account for differences in the class of animal (e.g., bulls, cows, steers, calves). Furthermore, the IPCC estimate used GWP values only for 100 years. The 20-year and 100-year GWP values based upon USA-specific emissions values provide greater precision and lower uncertainty (USEPA 2018). Therefore, these estimates are likely more accurate than those based on IPCC (2006) values.

Unsurprisingly, estimated emissions using the three approaches vary widely. For example, emissions from a single AUM range from 225 kg CO_2_e using conservative IPCC global default values to 875 kg CO_2_e using a GWP-20 and USA-specific values for cattle (Table 1). Most of the emissions arise from enteric fermentation with lesser amounts arising from manure deposition. The GWP-20 data suggest about 90% of the emissions comes from enteric emissions compared to about 80% using the GWP-100 data.

**Table 1:** The estimated annual greenhouse gas (GHG) emissions (kg) per animal unit month (AUM) arising from emissions of methane (CH_4_) and nitrous oxide (N_2_O) from enteric fermentation and manure deposition on rangelands

	CH_4_ emission/AUM	20 y GWP (CO_2_e)	100 y GWP (CO_2_e)	IPCC default (CO_2_e)
Methane emission fermentation	9.25	796	315	150
Methane emission manure	0.20	17	6.8	5.7
Total CH_4_ emission/AUM	9.45	813	321	156
N_2_O emission manure		63	70	70
Total GHG /AUM		875	391	225

GWP-20 are emissions based upon 20-year global warming potential; GWP-100 are based upon 100-year GWPs (IPCC 2013). Average methane emissions are for beef cows from USEPA (2018) except for IPCC default values which are from IPCC (2006). IPCC default values are also based upon a 100-year GWP

**Fig. 6:**
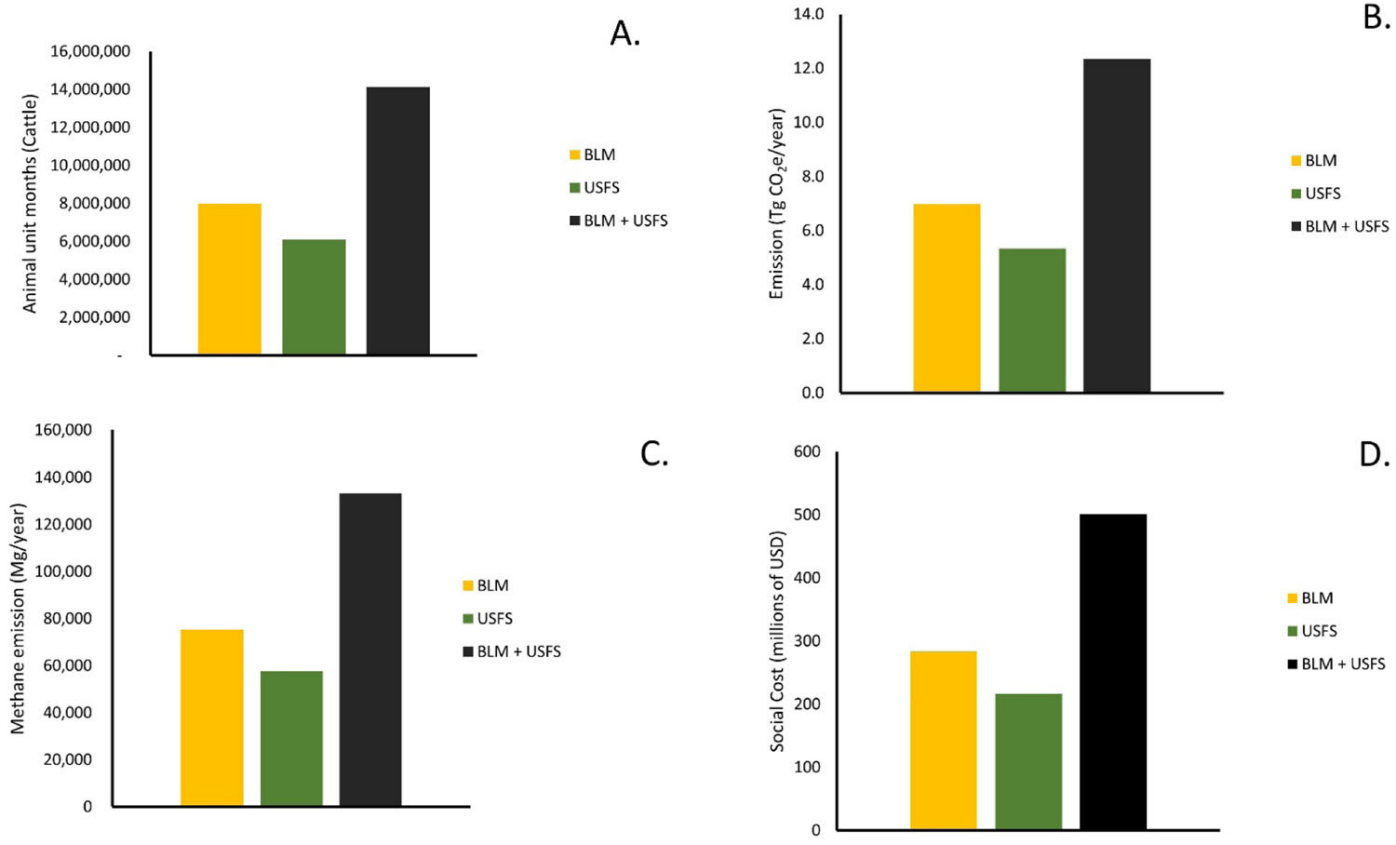
**A** The average number of animal unit months (AUMs) for cattle that utilized Bureau of Land Management (BLM) lands (2009–2018) and US Forest Service (USFS) lands in the western US (2007–2016). The totals (BLM + USFS) are means from the years 2009–2016. **B** The annual total emissions (Tg CO_2_e) from enteric fermentation and manure deposition on western public lands for the same time periods as above. **C** The annual total methane emissions (Mg) from cattle grazing public lands. **D** The annual social cost of carbon from livestock on public lands (millions of US dollars). The standard errors are not included as they were less than 2% of the mean (Supplementary Information, Table S3)

Livestock numbers on western public lands have not varied greatly in the past 10–20 years (Supplementary Information, Table S3; Glaser et al. 2015). A mean of 15.4 million AUMs of livestock use occurred annually from 2009–2016, and cattle account for over 91% of all domestic animals that graze BLM and USFS lands in the western USA. For the most recent 10-year period in which data are available, an average of 8.0 million AUMs of cattle grazed on public lands managed by the BLM and 6.1 million AUMs of cattle grazed USFS lands (Fig. 6A; Supplementary Information, Table S3).

Livestock grazing on BLM- and USFS-managed public lands generates significant quantities of greenhouse gases (Fig. 3B, C). Based upon the 20-year GWP, the mean GHG emissions from cattle on BLM-managed lands was 6.98 ± 0.06 Tg CO_2_e year^−1^. The mean GHG emissions from cattle on USFS-managed lands in the western US was 5.34 ± 0.09 Tg CO_2_e year^−1^. In total, about 12.35 ± 0.13 Tg CO_2_e year^−1^ arise from cattle grazing public lands in the western USA.

The annual emissions from enteric fermentation and manure deposition on western public lands are equivalent to the emissions from nearly 2.3 million passenger vehicles and are essentially equal to the emissions coming from all passenger vehicles in the western states of Idaho, Nevada, Utah, and Wyoming combined. These emissions are also equivalent to the amount of carbon that would be sequestered by 6.1 million ha of US forests (USEPA 2021). Emissions from methane alone are more than 133,000 Mg year^−1^ (Fig. 6C). Based upon a UNEP (2021) analysis of the effects of methane on the environment and societies, the reduction of methane emissions from removal of cattle on public lands in the western USA would avoid: 186 premature human deaths; 52 million hours of lost labor from extreme heat; and, 18,850 Mg of crop losses each year. In essence, allowing domestic livestock to graze public lands in the western USA results in declines in both human well-being and the productivity of other agricultural sectors. And again, cattle on public lands in the western USA account for <1.6% of all US beef production.

### The Social Cost of Carbon Related to Livestock Grazing on Public Lands Is Significant and Far Outweighs Modest Grazing Fee Payments Received by the USA

Recently, US federal agencies have recognized that it is essential for them to capture the full costs of greenhouse gas emissions as accurately as possible, including by taking global damages into account (e.g., Executive Order 13990 (2021) and Interior Secretarial Order 3399 2021). The social cost of carbon (SCC) is a central concept for understanding, evaluating, and implementing climate change policies. The SCC is an estimate of the monetized damages associated with incremental increases in greenhouse gas emissions. It represents the present value of the marginal social damages of increased GHG emissions in a particular year—including the impacts of global warming on agricultural productivity and human health, loss of property and infrastructure to sea level rise and extreme weather events, diminished biodiversity and ecosystem services, etc.—and therefore it also represents the marginal social benefits of emissions reductions.

The SCC (carbon dioxide) was $51/Mg in 2020 with methane and nitrous oxide emission costs at $1,500/Mg and $18,000/Mg, respectively (Interagency Working Group on Social Cost of Greenhouse Gases 2021). These costs are expected to rise to $85/Mg for carbon dioxide, $3,100/Mg for methane, and $33,000/Mg for nitrous oxide by 2050. The social costs presented here are based on 2020 values.

The SCC for greenhouse gas emissions from cattle was calculated from four different data sets (Table 2). Nitrous oxide and methane costs were calculated from the social cost assigned to these gases. The GWP-20, GWP-100, and IPCC default values arise from the calculated greenhouse gas emissions on a carbon dioxide equivalence basis.

**Table 2:** The social cost ($USD) per animal unit month (AUM) of methane (CH_4_), nitrous oxide (N_2_O), and carbon (CO_2_e) arising from the enteric fermentation and manure deposition of cattle on rangelands

	N_2_O and CH_4_	GWP-20	GWP-100	IPCC default
Methane emission—fermentation	$28.68	$40.57	$16.04	$7.66
Methane emission—manure deposition	$2.62	$0.88	$0.35	$0.29
Subtotal social cost CH_4_ emission/AUM	$31.30	$41.45	$16.39	$7.95
N_2_O emission—manure	$4.20	$3.19	$3.55	$3.55
Total social cost/AUM	$35.50	$44.64	$19.93	$11.49

The N_2_O–CH_4_ costs are based upon the social cost of N_2_O and CH_4_ while GWP-20, GWP-100, and IPCC defaults are based upon the social cost of carbon (CO_2_e). Data are based upon values determined at a 3% discount rate which is $1500/metric ton for CH_4_, $18,000/metric ton for N20, and $51 per metric ton for CO_2_e (Interagency Working Group on Social Cost of Greenhouse Gases, United States Government 2021). Calculations of the social costs reported in this text use the N_2_O and CH_4_ costs

Depending upon the approach used, the social costs of the greenhouse gases from cattle grazing on western US public lands range from about $11 to $45 per AUM (Table 2). The most direct estimate entails using the nitrous oxide and methane emission costs and is therefore suggested to be the estimate with the least uncertainty. Using this approach, the social cost of greenhouse gas emission for a single AUM is approximately $36/AUM.

The social costs of emissions from greenhouse gases from enteric fermentation and manure deposition from western public lands grazing averaged $501 million per year from 2010–2016 (Fig. 6D; Supplementary Information, Table S3). These social costs do not include the values of carbon gain via sequestration if the lands were no longer grazed by cattle. It is probable that the values associated with the lost potential of carbon sequestration due to livestock impacts would be even greater than the benefits from the elimination of emissions via enteric fermentation. Determination of carbon sinks, emissions, and sequestration from public lands would be difficult given the vast area of land involved coupled with the large numbers of cattle that are contributing to, and exacerbating climate change. But the increased carbon storage potential would be great. For example, we predicted that the carbon that could be sequestered though cessation of livestock grazing in riparian areas could be 2.2 Tg C/year (8.1 million Tg CO_2_e/year). This is a SCC value of $413 million per year. An estimated 24.7 to 80.2 Tg CO_2_e have been lost through purposeful conversion to exotic-dominated grasslands (i.e., a SCC of $1.3 - 4.0 billion). The carbon losses associated with type conversion to cheatgrass dominance would be at least 268.5 Tg CO_2_e (a SCC of $13.7 billion). Shifting public lands from sources of greenhouse gases to carbon sinks could be quickly attained via the removal of livestock grazing.

### Without Public Lands Grazing, Wouldn’t there Be Leakage?

An argument for maintaining livestock grazing on public lands is that if cattle are not using these areas, they will be grazing somewhere else and hence there is no net loss of greenhouse gas emissions (the concept of leakage). But this argument ignores the carbon potentially gained via increased sequestration and storage on public landscapes if they are ungrazed by cattle. Such a change in public lands management would result in a net increase in carbon removals with little leakage.

Forage quality is a strong determinant of the amount of methane produced by ruminants. Sources of forage with a relatively low digestible energy content will produce relatively high quantities of methane. For example, crested wheatgrass and annual bromes are forages with notably low digestible energy contents, only 58 and 53%, respectively (USEPA 2018). Furthermore, late in the grazing season (e.g., August–October) these dried grasses will have digestible nutrient concentrations like that of straw (a digestible energy content of about 39%), suggesting that cattle on these diets would emit higher quantities of methane than on a diet of forages with high digestible energy. This is why methane emissions from feedlot cattle are only 35–43 kg CH_4_ year^−1^, compared to 89–95 kg CH_4_ year^−1^ for cattle on rangelands (USEPA 2018, Supplementary Information, Table S2). Thus, substituting the relatively poor quality of forages on rangelands, especially degraded rangelands, with higher quality feeds from other sources would represent a net reduction in greenhouse gas emissions (UNEP 2021). For this reason, the forage from public lands, especially when high in exotic grasses, is about the worst diet to feed cattle from a greenhouse gas perspective. Achieving very low emissions from the production of edible animal proteins may involve large-scale industrialized agriculture, which can have other social and environmental impacts beyond greenhouse gas emissions and hence such policies need to be considered with care (UNEP 2021). Dietary shifts away from beef would significantly contribute to reducing greenhouse gas emissions (Clark et al. 2019; Springmann et al. 2018).

### The True Cost of Grazing Public Lands

The federal grazing fee for 2020 and 2021, set by a formula established by Congress in 1978, is $1.35 per AUM for public lands managed by the BLM and USFS (USDI-BLM 2021). In contrast, the estimated social cost of greenhouse gases arising from a cow-calf pair on public lands is nearly $36 (Table 2), or 26 times greater than the federal grazing fee. Furthermore, the administrative costs for managing livestock grazing on public lands have been estimated to range from approximately $8-$12 per AUM (GAO 2005; Glaser et al. 2015). Thus, the total costs to the US taxpayers and society for grazing a single AUM on public land may be at least $42–$48. Combining management costs with social costs of greenhouse gases from the more than 14 million AUMs of livestock that graze public lands in the western USA results in a total cost to taxpayers exceeding $608 million each year.

We limited our analyses to: (1) the greenhouse gas emissions from domestic livestock enteric fermentation and manure deposition while grazing public lands; (2) potential changes in carbon stocks due to grazing in the widespread sagebrush biome; and (3) the effects of grazing and livestock management on carbon sequestration and greenhouse gas emissions from these ecosystems on public lands. We did not examine in detail other important considerations that would be essential to calculate the true cost of grazing public lands. First, it is important to note that this is not a complete accounting of the greenhouse gas emissions associated with domestic cattle grazing on public lands (i.e., a life cycle analysis). For example, not included in this analysis are activities such as trucking livestock to and from private lands and to meat processing facilities, the costs of fencing, maintenance of water developments and hauling mineral supplements and water (which may increase with climate change), rangeland seeding and invasive species management, and many other ecological, economic and carbon costs associated with public lands grazing. In addition, the greenhouse gas emissions arising from the administration and monitoring of grazing permits were not included. Second, it is important to note this is not a complete accounting of the potential changes in carbon stocks due to grazing. For example, this analysis focuses on the loss of above ground carbon and does not quantify the potential significant loss of below ground biomass and biological soil crusts as a result of livestock grazing (Beschta et al. 2012; Bradley et al. 2006). Last, we did not ascertain social costs of desertification from overgrazing, losses in water quality and quantity, losses in biodiversity, losses in carbon sequestration capacity of the landscape, and the other ecosystem services negatively affected by livestock grazing. In short, the carbon sequestration losses and greenhouse gas emissions presented in this paper, while significant, nevertheless underestimate, perhaps substantially, the true costs of livestock grazing western public lands.

## Conclusions

Improved stewardship of public lands in the western US is needed to achieve the international Paris Agreement on climate change and the USA’s goals of reducing emissions and holding warming to below 2 °C. Nature-based or natural climate solutions include the conservation, restoration, and/or improved land management actions that increase carbon storage and/or avoid greenhouse gas emissions across global forests, wetlands, grasslands, and agricultural lands (Griscom et al. 2017). Given their vast area, significant carbon stocks, large extent of degradation, and high levels of greenhouse gas emissions associated with livestock grazing, the public lands in the western USA can play an important role in meeting government policy goals and addressing the climate crisis.

Land degradation, including loss of native vegetation, annual grass invasion, devastating fires, and losses of major carbon sinks is a heavy price to pay for the minimal economic gains from use of these intrinsically unproductive lands for livestock production (Meyer 2011). Grazing exclusion is an effective ecosystem restoration approach to sequester and store carbon in the living biomass and soil profiles, and hence, an important tool for climate change mitigation (Reda 2018). Removing livestock can increase soil carbon sequestration on lands that have been depleted in the past by poor management. Removing livestock is not only a viable, cost-effective natural climate solution; it also offers enhanced water quality, flood buffering, soil health, habitat diversity, and climate resilience (Beschta et al. 2012). Compensating holders of federally-issued grazing permits who wish to voluntarily relinquish their permits to graze public lands could accelerate the process and confer additional, complimentary economic, social and environmental benefits (Leshy and McUsic 2008; Salvo and Kerr 2006).

The United States has announced a target for achieving a 50–52% reduction from 2005 levels in economy-wide net greenhouse gas pollution by 2030, and a net-zero emissions economy by 2050. Attaining net-zero emissions requires transformative action across all sectors of society including the agricultural and natural resource sectors. To achieve these goals all federal and state agencies will need to contribute, and those entrusted to manage public lands are no exception. Outdated approaches to public land management are in conflict with stated current US climate goals, as these actions often increase greenhouse gas emissions, lower the carbon sequestration capacity, and increase the vulnerability of the public resources. Yet, changes in federal land management policy offer a significant opportunity for building climate resiliency where ungrazed landscapes are net carbon sinks of greenhouse gases within some of the most biologically diverse, expansive, and vulnerable ecosystems in North America.

## Supplementary information

Supplementary Information

## Data Availability

Data on the aboveground biomass and carbon stocks of dominant semiarid ecosystems can be found in the Supplementary Information. Data on the numbers of livestock may be found at online databases provided by the USDA Forest Service (2021) and the USDI Bureau of Land Management (2021). Data on emissions from livestock in the USA may be found in US Environmental Protection Agency (2018). Global default values of methane emissions from enteric fermentation for beef cattle are from IPCC (2006).
