# Supplementary material for: Livestock Use on Public Lands in the Western USA Exacerbates Climate Change: Implications for Climate Change Mitigation and Adaptation
Source: Environ Manage. 2022 Apr 2;69(6):1137–52. doi: 10.1007/s00267-022-01633-8 (PMC9079022; doi:10.1007/s00267-022-01633-8)
Supplement: Supplementary file 1 — Supplementary Information [file 267_2022_1633_MOESM1_ESM.docx]

Supplementary Information: Livestock on public lands of the western USA accentuate effects of climate change: Implications for mitigation and adaption

J. Boone Kauffman

Robert L. Beschta

Peter M. Lacy

Marc Liverman

| Supplementary Information, Table S1. Total aboveground biomass (Mg/ha) and total carbon stocks (Mg C/ha) for intact big sagebrush communities, cheatgrass and crested wheatgrass stands. Aboveground pools include living and dead mass including litter. | | | | | |
| --- | --- | --- | --- | --- | --- |
|  | Total aboveground biomass | | Total aboveground carbon stock* | Location | Citation |
| A tridentata-wyomingensis | 6.01 (0.8) | 2.7 | | Hart Mountain NAR, Oregon | Reis et al. (2018) |
| A tridentata-wyomingensis (late seral) | 5.6 (0.6) | 2.5 | | Hart Mountain NAR | Ellsworth and Kauffman (2017) |
| A tridentata-wyomingensis (mid- seral) | 1.6 | 0.7 | | Hart Mountain NAR | Ellsworth and Kauffman (2017) |
| A tridentata-wyomingensis (early seral) | 1.05 (0.03) | 0.47 | | Hart Mountain NAR | Ellsworth and Kauffman (2017) |
| A tridentata - tridentata | 10.59 (1.12) | 4.8 | | John Day Fossil Beds, Oregon | Sapsis and Kauffman (1991) |
| A tridentata - tridentata | 6.23 ().69) | 2.8 | | John Day Fossil Beds, Oregon | Sapsis and Kauffman (1991) |
| A tridentata - tridentata | 8.2 (1.0) | 3.7 | | John Day Fossil Beds, Oregon | Ellsworth et al. (2000) |
| A tridentata - tridentata | 28.85 | 13.0 | | Bear Creek, Oregon | Kauffman and Cummings (1989) |
| A tridentata - tridentata | 25.84 | 11.6 | | Bear Creek, Oregon | Kauffman and Cummings (1989) |
| A tridentata – vaseyana | 6.55 | 2.95 | | Lava Beds National Mon., California | Ellsworth and Kauffman (2010) |
| A tridentata – vaseyana | 6.89 | 3.10 | | Lava Beds National Mon., California | Ellsworth and Kauffman (2010) |
| A tridentata – vaseyana | 8.98 | 4.0 | | Lava Beds National Mon., California | Ellsworth and Kauffman (2010) |
| A tridentata – vaseyana | 9.98 | 4.45 | | Lava Beds National Mon., California | Ellsworth and Kauffman (2010) |
| A tridentata – vaseyana | 21.62 | 9.7 | | Lava Beds National Mon., California | Ellsworth and Kauffman (2010) |
| A. tridentata – vaseyana (early seral) | 1.69 (0.65) | 0.8 | | Hart Mountain NAR | Ellsworth and Kauffman (2017) |
| A. tridentata – vaseyana (mid seral) | 9.4 (4.8) | 4.2 | | Hart Mountain NAR | Ellsworth and Kauffman (2017) |
| A. tridentata – vaseyana (late seral) | 11.57 (1.81) | 5.2 | | Hart Mountain NAR | Ellsworth and Kauffman (2017) |
| A. tridentata – vaseyana (very late seral) | 25.9 (3.6) | 11.7 | | Hart Mountain NAR | Ellsworth and Kauffman (2017) |
| A. arbuscula (early seral) | 0.59 | 0.3 | | Hart Mountain NAR | Ellsworth and Kauffman (2017) |
| A. arbuscula (mid seral) | 2.97 (0.77) | 1.3 | | Hart Mountain NAR | Ellsworth and Kauffman (2017) |
| A. arbuscula (late seral) | 4.32 (0.78) | 1.9 | | Hart Mountain NAR | Ellsworth and Kauffman (2017) |
| Bromus tectorum (Cheatgrass) | 0.5-1.2 | 0.2-0.5 | | Northwestern Nevada | Diamond et al (2009) |
| Bromus tectorum | 0.20.-0.9 | 0.1-0.4 | | Northcentral Nevada | Bradley et al. (2006) |
| Bromus tectorum | 0.45 | 0.2 | | NE Oregon | Wright et al. (2012) |
| Agropyron cristatum | 1.1 | 0.5 | | SE Oregon | Wright et al. (2012) |
| Juniperus occidentalis woodland | 49.91 | 22.46 | | Central Oregon | Abdallah et al. (2020) |
| Juniperus occidentalis woodland | 31.5 | 14.18 | | Central Oregon | Kauffman and Cummings (1989) |
| Average A. tridentata-wyomingensis | 5.8 | 2.6 | |  |  |
| Average A. tridentata-tridentata | 17.3 | 7.8 | |  |  |
| Average A. tridentata-vaseyana | 12.98 | 5.8 | |  |  |
| Average B. tectorum | 0.5 | 0.23 | |  |  |
| Dry biomass was converted to aboveground carbon using a C/biomass conversion factor of 0.45. | | | | | |

| Supplementary Information, Table S2. Methane and nitrous oxide emissions (kg C/animal unit/year) arising from enteric fermentation in beef cattle. Unless otherwise noted the USEPA measures for Oregon are used to estimate greenhouse gas emissions from cattle. Total enteric emission from a cow calf operation would total 111 kg/yr. Manure emissions from the same cow-calf operation is assumed to total emissions from 2 animals. | | | | | | | | | | |  | |  |
| --- | --- | --- | --- | --- | --- | --- | --- | --- | --- | --- | --- | --- | --- |
| Animal class | Enteric  fermentation | | Manure  emissions | N_2_0 emissions  from manure | | | | | | | Source | |  |
| Default global value – each individual animal | 53 | 1 | | | 1.4 |  | |  | | | | IPCC (2006) | |
| Default global value – each individual animal | 58.5 | 2.4 | | |  |  |  | | | | | Wolf et al. (2017) | |
| Default global value – each individual animal | 75 | 1.7 | | |  | | | |  |  | | USEPA (2018) | |
| Cows (national default value) | 95 |  | | |  | | | |  |  | | USEPA (2018) | |
| Calves (national default value) | 11 |  | | |  | | | |  |  | | USEPA (2018) | |
| Stocker steers (national default value) | 58 |  | | |  | | | |  |  | | USEPA (2018) | |
| Cows (Western US states) | 100 |  | | |  | | | |  |  | | USEPA (2018) | |
| Calves (Western US states) | 11 |  | | |  | | | |  |  | | USEPA (2018) | |
| Stocker steers (Western US states) | 58 |  | | |  | | | |  |  | | USEPA (2018) | |
| Feedlot cattle (national default value) | 43 |  | | |  | | | |  |  | | USEPA (2018) | |
| Feedlot cattle (Western US states) | 35 |  | | |  | | | |  |  | | USEPA (2018) | |

| Supplementary Information, Table S3. Livestock numbers, expressed as animal unit months (AUMs), for cattle and all livestock on public lands in the American West and the greenhouse gas emissions (Tg CO_2_e) arising from their enteric fermentation and manure deposition. AUM data are from USDA( <https://www.fs.fed.us/rangeland-management/reports/index.shtml>) and USDI grazing statistics (<https://www.blm.gov/about/data/public-land-statistics> and accessed 1 July 2021) for the reported years. Slight variations in numbers may occur as federal databases are frequently revised. | | | | | | | | | | |  |
| --- | --- | --- | --- | --- | --- | --- | --- | --- | --- | --- | --- |
|  | BLM | | | US Forest Service - Western USA | | | Total AUMs | | | | |
| Year | Cattle (AUMS) | All livestock (AUMs) | Emissions (Tg) | Cattle (AUMs) | All livestock (AUMS) | Emissions (Tg) | Cattle (AUMs) | All livestock (AUMS) | Emissions (Tg) | Emissions (Tg) | |
| 2007 |  |  |  | 5,382,546 | 5,925,122 | 4.71 |  |  |  |  | |
| 2008 |  |  |  | 6,029,319 | 6,626,498 | 5.28 |  |  |  |  | |
| 2009 | 7,861,357 | 8,608,534 | 6.88 | 6,101,892 | 6,706,883 | 5.34 | 13,963,249 | 15,315,417 | 0.91 | 12.22 | |
| 2010 | 8,040,441 | 8,763,115 | 7.04 | 6,450,012 | 7,089,257 | 5.64 | 14,490,453 | 15,852,372 | 0.91 | 12.68 | |
| 2011 | 8,245,132 | 8,997,890 | 7.21 | 6,219,446 | 6,831,975 | 5.44 | 14,464,578 | 15,829,865 | 0.91 | 12.66 | |
| 2012 | 7,953,707 | 8,665,594 | 6.96 | 6,325,674 | 6,938,616 | 5.53 | 14,279,381 | 15,604,210 | 0.92 | 12.49 | |
| 2013 | 7,530,535 | 8,231,894 | 6.59 | 5,833,612 | 6,422,424 | 5.10 | 13,364,147 | 14,654,318 | 0.91 | 11.69 | |
| 2014 | 7,637,592 | 8,331,350 | 6.68 | 6,003,258 | 6,568,274 | 5.25 | 13,640,850 | 14,899,624 | 0.92 | 11.94 | |
| 2015 | 8,027,200 | 8,703,768 | 7.02 | 6,317,407 | 6,891,646 | 5.53 | 14,344,607 | 15,595,414 | 0.92 | 12.55 | |
| 2016 | 8,055,874 | 8,751,729 | 7.05 | 6,326,655 | 6,894,148 | 5.54 | 14,382,529 | 15,645,877 | 0.92 | 12.58 | |
| 2017 | 8,118,945 | 8,811,353 | 7.10 |  |  |  |  |  |  |  | |
| 2018 | 8,350,907 | 9,052,358 | 7.31 |  |  |  |  |  |  |  | |
| Mean | 7,982,169 | 8,691,759 | 6.98 | 6,098,982 | 6,689,484 | 5.34 | 14,116,224 | 15,424,637 | 0.92 | 12.35 | |
| SE | 79,777 | 81,368 | 0.07 | 99,241 | 105,382 | 0.09 | 147,968 | 154,601 | 0.00 | 0.13 | |

| Supplementary Information, Table S4. The social costs ($USD) of carbon related to the emissions arising from enteric fermentation and manure deposition on Bureau of Land Management (BLM) and US Forest Service (USFS) managed lands in the American West. Data are based upon the most recent livestock numbers available for these public lands. The social cost is based upon costs from combined CH_4_ and N_2_O emissions per AUM ($35.50/AUM; Table 2). | | | |
| --- | --- | --- | --- |
| Year | BLM | USFS | BLM and USFS |
| 2007 |  | $191,080,383 |  |
| 2008 |  | $214,040,825 |  |
| 2009 | $279,078,174 | $216,617,166 | $495,695,340 |
| 2010 | $285,435,656 | $228,975,426 | $514,411,082 |
| 2011 | $292,702,186 | $220,790,333 | $513,492,519 |
| 2012 | $282,356,599 | $224,561,427 | $506,918,026 |
| 2013 | $267,333,993 | $207,093,226 | $474,427,219 |
| 2014 | $271,134,516 | $213,115,659 | $484,250,175 |
| 2015 | $284,965,600 | $224,267,949 | $509,233,549 |
| 2016 | $285,983,527 | $224,596,253 | $510,579,780 |
| 2017 | $288,222,548 |  |  |
| 2018 | $296,457,199 |  |  |
|  |  |  |  |
| Mean | $283,367,000 | $216,513,865 | $501,125,961 |
| SD | $8,955,832 | $11,140,904 | $14,857,362 |
| SE | $2,832,083 | $3,523,063 | $5,252,871 |

| \| Supplementary Information, Table S5. US beef cattle numbers, average weights and animal unit equivalents in 2015. Data are from the USEPA (2018). Numbers do not include dairy cattle. \| \| \| \| \| \| \| \| \| \| --- \| --- \| --- \| --- \| --- \| --- \| --- \| --- \| --- \| \| Cattle Type \| Number (x1000) \| Avg Wt. (lbs) \| Avg Wt. (kg) \| No. per animal unit \| AUs \| AUMs \| Total AUMs (x1000) \| \| \| Calves \| 15,117 \| 270 \| 123 \| 2.7 \| 0.4 \| 4.5 \| 68,184 \| \| \| Bulls \| 2,109 \| 2022 \| 918 \| 0.6 \| 1.7 \| 20.4 \| 43,063 \| \| \| Cows \| 29,302 \| 1348 \| 612 \| 0.8 \| 1.3 \| 15.1 \| 441,427 \| \| \| Beef replacements (7-11 mons) \| 1,473 \| 891 \| 404 \| 1.1 \| 0.9 \| 11.0 \| 16,267 \| \| \| Beef replacements 12-23 mon) \| 3,422 \| 891 \| 404 \| 1.1 \| 0.9 \| 11.0 \| 37,790 \| \| \| steer stockers \| 7,517 \| 717 \| 325 \| 1.3 \| 0.8 \| 9.4 \| 70,531 \| \| \| Heifer stockers \| 4,402 \| 713 \| 324 \| 1.3 \| 0.8 \| 9.3 \| 41,130 \| \| \| Feedlot cattle \| 12,883 \| 980-1037 \| 680 \| 0.7 \| 1.4 \| 16.3 \| 210,065 \| \| \| Total beef cattle \| 76,225 \|  \|  \|  \|  \|  \| 928,458 \| \| \| Total beef cattle - calves \| 61,108 \|  \|  \|  \|  \|  \| 860,274 \| \| \| Total cattle AUMS on public lands = 14,116224 \| \| \| \| \| \| \| \| \| \| \| Percent of forage from public lands - cows only = 3.2% \| \| \| \| \| \| \| \| \| \| \| Percent of forage from public lands - all beef cattle excluding calves = 1.6% \| \| \| \| \| \| \| \|  \| \| |  |  |  |  |  |  |
| --- | --- | --- | --- | --- | --- | --- | --- | --- | --- | --- | --- | --- | --- | --- | --- | --- | --- | --- | --- | --- | --- | --- | --- | --- | --- | --- | --- | --- | --- | --- | --- | --- | --- | --- | --- | --- | --- | --- | --- | --- | --- | --- | --- | --- | --- | --- | --- | --- | --- | --- | --- | --- | --- | --- | --- | --- | --- | --- | --- | --- | --- | --- | --- | --- | --- | --- | --- | --- | --- | --- | --- | --- | --- | --- | --- | --- | --- | --- | --- | --- | --- | --- | --- | --- | --- | --- | --- | --- | --- | --- | --- | --- | --- | --- | --- | --- | --- | --- | --- | --- | --- | --- | --- | --- | --- | --- | --- | --- | --- | --- | --- | --- | --- | --- | --- | --- | --- | --- | --- | --- | --- | --- | --- | --- | --- | --- | --- | --- | --- | --- | --- | --- | --- | --- | --- | --- | --- | --- | --- | --- | --- | --- | --- | --- |

Literature cited

# Abdallah MAB, Mata-González R, Noller, JS, Ochoa CG (2020) Ecosystem carbon in relation to woody plant encroachment and control: Juniper systems in Oregon, USA. *Agriculture, Ecosystems & Environment*, 290, 106762, <https://doi.org/10.1016/j.agee.2019.106762>

Bradley BA, Houghton RA, Mustard JF, Hamburg SP (2006) Invasive grass reduces aboveground carbon stocks in shrublands of the western US. *Global Change Biology,* **12**, 1815-1822.

Ellsworth LM, Kauffman JB (2010) Native bunchgrass response to prescribed fire in ungrazed mountain big sagebrush ecosystems. *Fire Ecology* 6:86-96.

Ellsworth LM, Kauffman JB (2017) Long-term impacts of wildfire on fuel loads, vegetation composition, and potential fire behavior and management in sagebrush-dominated ecosystems. *Final Report for Joint Fire Science Program Project 14-1-02-5*, Boise, ID, <https://www.firescience.gov/projects/14-1-02-5/project/14-1-02-5_final_report.pdf> 21p.

Ellsworth LM, Kauffman JB, Reis SA, Sapsis DB, Moseley K (2020). Repeated fire altered succession and increased fire behavior in basin big sagebrush–native perennial grasslands. *Ecosphere* 11(5): e03124. 10.1002/ecs2.3124.

IPCC (Intergovernmental Panel on Climate Change) (2006) Guidelines for national greenhouse gas inventories, Chapter I. Introduction. Prepared by the National Greenhouse Gas Inventories Programme, Eggleston HS, Buendia L, Miwa K, Ngara T, Tanabe K(eds) Published: IGES, Japan, pp:1-21.

Kauffman JB and DL Cummings (1989) Fuel loads and biomass consumption during spring and fall prescribed fires in central Oregon rangeland ecosystems. Unpublished report on file at the U. S. Department of Agriculture, Forest Service, Pacific Northwest Research Station, Pacific Wildland Fire Sciences Laboratory, Seattle, WA.

Reis SA, Ellsworth LM, Kauffman JB, Wrobleski D (2019) Long-term effects of fire on vegetation structure and predicted fire behavior in Wyoming Big Sagebrush ecosystems. *Ecosystems*, **22**, 257–265.

Sapsis DB, Kauffman JB (1991) Fuel consumption and fire behavior associated with prescribed fires in basin big sagebrush-dominated ecosystems. *Northwest Science* 65:173-179.

United States Department of Agriculture (2021). Grazing Statistical Summary 2006-2017. <https://www.fs.fed.us/rangeland-management/reports/index.shtml>. Accessed 1 July 2021.

United States Department of Interior. Bureau of Land Management (2021) Public lands Statistics 2009-2018, <https://www.blm.gov/about/data/public-land-statistics>. Accessed 1 July 2021.

United States Environmental Protection Agency (2018) A-250. Inventory of U.S. Greenhouse Gas Emissions and Sinks: 1990-2016. *United States Environmental Protection Agency*, <https://www.epa.gov/sites/production/files/2018-01/documents/2018_annex_3_-_part_b.pdf>. Accessed 15 July 2021

Wolf J, Asrar GR, West TO (2017) Revised methane emissions factors and spatially distributed annual carbon fluxes for global livestock. *Carbon Balance Manage* **12,** 16, <https://doi.org/10.1186/s13021-017-0084-y>

Wright CS, Vihnanek RE, Restaino JC, Dvorak JE (2012) Photo series for quantifying natural fuels. Volume XI: eastern Oregon sagebrush-steppe and northern spotted owl nesting habitat in the Pacific Northwest. *USDA Forest Service, General Technical Report*, PNW-GTR-878, Portland, OR, 85 p.
